# Defocus-Incorporated Multiple-Segment (DIMS) Spectacle Lenses Versus Single-Vision Lenses for Myopia Control in a South-Eastern European Population

**DOI:** 10.3390/bioengineering13070802

**Published:** 2026-07-13

**Authors:** Valeria Mocanu, Raluca Horhat, Florin-Raul Horhat, Mihai Poenaru-Sava, Mihnea Munteanu

**Affiliations:** 1 Ophthalmology Department, “Victor Babes” University of Medicine and Pharmacy, 300041 Timisoara, Romania; mocanu.valeria@umft.ro (V.M.); mihai.poenaru-sava@umft.ro (M.P.-S.); mihnea.munteanu@umft.ro (M.M.); 2 Ophthalmology Clinic, Timisoara Municipal Emergency Hospital, 300254 Timisoara, Romania; 3 Ophthalmo-ENT Tumor Sensory Research Center (EYE-ENT), “Victor Babes” University of Medicine and Pharmacy, 300041 Timisoara, Romania; 4 Department of Functional Sciences, “Victor Babes” University of Medicine and Pharmacy, 300041 Timisoara, Romania; 5 Clinic of Pediatric Surgery, Emergency Children’s Hospital Louis Turcanu, 300011 Timisoara, Romania; 6 Center for Modeling Biological Systems and Data Analysis, “Victor Babes” University of Medicine and Pharmacy, 300041 Timisoara, Romania; 7 Department of Mathematics, Polytechnic University of Timisoara, 300006 Timisoara, Romania; rhorhat@umft.ro

**Keywords:** myopia, DIMS lenses, axial length, pediatric population

## Abstract

Background: Myopia is a refractive error reaching alarmingly high levels worldwide and is associated with severe complications such as: maculopathy, retinal detachment, open-angle glaucoma, and posterior subcapsular cataract. The study compared two treatment strategies: defocus-incorporated multi-segment (DIMS) spectacle lenses and single-vision (SV) lenses. Methods: 201 children (mean age 10.92 ± 2.11 years, range, 7–17 years) were included in the study (104 patients, DIMS group; 97 patients, SV group). The ophthalmological examination included best corrected visual acuity (BCVA), cycloplegic refractive measurement (SER), eye movement evaluation, cover test, prism cover-uncover tests for near and distance, slit-lamp evaluation, fundus examination and ocular biometry. Results: The mean SER progression in the right eye over the 12-month follow-up was −0.29 ± 0.3 D in the DIMS group and −0.58 ± 0.45 in the SV group. Children wearing DIMS exhibited significantly less myopia progression (*p* < 0.001). The multiple linear regression model showed no significant effect of sex or baseline axial length (AL) on SER change (*p* = 0.057 and 0.17, respectively), whereas treatment lens, age at diagnosis, provenance and baseline SER had a significant effect on SER change (*p* < 0.001). Conclusions: DIMS lenses are an effective treatment in myopia management in the Caucasian population.

## 1. Introduction

Refractive errors, defined as an imbalance among the eye’s most important optical components—the cornea, the lens, and the axial length (AL)—are common ocular disorders. During infancy and childhood, the refractive power of the cornea and lens decreases, while the axial length progressively increases. When the AL elongation exceeds the eye’s focal point, myopia develops [1]. 

Myopia is reaching alarmingly high levels worldwide [2,3,4]. In East and Southeast Asia, 70–80% of young adults are myopic, and approximately 20% of myopic children have a refractive error worse than −6 diopters (D) [2,3,4]. In Europe, the highest prevalence was reported in Sweden (49.67%), followed by Russia (46.17%) [5,6]. Although no epidemiological data are currently available for Romania, Bulgaria, a neighboring country, has reported a prevalence ranging from 7.83% to 16.85% [6]. The higher prevalence of myopia observed in many parts of Asia may be explained by earlier academic demands and higher levels of urbanization.

High myopia, defined by spherical equivalent (SER) ≤ −6 D and/or AL ≥ 26 mm, is associated with an increased risk of severe complications such as myopic maculopathy, retinal detachment, open-angle glaucoma, and posterior subcapsular cataract [1,7,8].

The onset of myopia usually occurs before the age of 10, with progression during teenage years and early twenties [1]. By 2050, it is estimated that half of the world population will be affected by myopia, with high myopia accounting for approximately 10% of the cases [9,10]. 

The pathogenic mechanisms underlying the development of myopia remain unclear. Recent studies suggest that it results from a combination of risk factors, including family history of myopia, ethnicity, socioeconomic status (e.g., education and occupation), near-work and outdoor activities, lens alterations and ocular dimensions [11,12,13,14,15].

The Correction of Myopia Evaluation Trial (COMET) study defined progressive myopia in children aged 6 to 12 years as an increase of more than −0.75 diopters (D) per year [16]. Other studies have reported a mean annual myopia progression rate of −0.55 D in Europe and −0.82 D in the Asian population [17].

Myopia management consists of three main approaches: lifestyle modifications, the use of atropine (ATP) and various optical correction lenses [18,19]. Among the most commonly used optical correction options are soft and rigid contact lenses, orthokeratology contact lenses, bifocal spectacles, progressive addition lenses (PALs), bifocal and prismatic bifocal spectacles and defocus-incorporated multi-segment (DIMS) spectacles [20,21,22,23,24]. All of these options have advantages and disadvantages, as well as varying levels of effectiveness in slowing myopia progression; however, none has been shown to completely halt its progression [25,26]. High-concentration ATP eye drops (1%) have been reported to be highly effective in reducing the rate of myopia progression. However, due to its side effects such as cycloplegia, pupil dilation, and impairment of visual function, lower concentrations are generally recommended (e.g., 0.01%) [25,26,27,28]. Recent clinical trials have shown that low-concentration (0.01%) ATP eye drops are associated with minimal side effects but provide only modest treatment efficacy with a low degree of myopic rebound after discontinuation [25,26,27,28,29,30].

Orthokeratology improves unaided daytime visual acuity; however, it may increase higher-order aberrations and reduce low-contrast visual acuity (VA) [31,32,33]. Decreased accommodation has been reported with the use of PALs, bifocal spectacles and multifocal soft contact lenses [34,35,36].

In the last decade, several studies have reported promising results with DIMS spectacle lenses in slowing myopia progression [37]. DIMS lenses consist of a central optical zone for distance vision correction, and an annular mid-peripheral zone containing multiple segments with a +3.50 D addition [38]. The combination of clear central vision and peripheral myopic defocus is thought to modulate scleral remodeling and reduce axial elongation by inducing the release of retinal neurotransmitters [39,40].

DIMS lenses are plastic spectacle lenses using a unique dual-focus design to generate two simultaneous optical signals to the eye. A central circular zone, measuring 9–10 mm in diameter, corrects the distance refractive error and allows light to focus directly on the retina, providing clear vision. The central optical zone is surrounded by a ring of hundreds of 1 mm “lenslets” with a relative positive power of +3.50 D. Therefore, peripheral light is focused in front of the retina, resulting in blurred retinal images—a state known as myopic defocus [28].

The aim of the present study was to compare myopia progression in a South-Eastern European pediatric population wearing DIMS lenses versus SV lenses over a 1-year period.

## 2. Materials and Methods

### 2.1. Study Group

This retrospective cohort study was conducted at the Ophthalmology Department of “Victor Babes” University of Medicine and Pharmacy in Timisoara, Romania. The study adhered to the principles of the Declaration of Helsinki, and, due to its retrospective nature, the institutional ethics committee waived the requirement for specific approval (no. 95/2025). All patients who undergo ophthalmological examinations and/or surgical procedures provide explicit informed consent for the use of their data in research. Patient confidentiality was maintained at all times throughout the study.

The medical records of children with myopia undergoing spectacle lens treatment between January 2023 and December 2025 were reviewed. Two treatment strategies were included in the study: DIMS lenses alone or SV lenses alone. The inclusion criteria consisted of the following: (1) age under 18 years; (2) myopia with a spherical equivalent refraction between −0.50 and −6.00 D; (3) astigmatism and anisometropia up to 1.50 D; (4) monocular best corrected visual acuity (BCVA) of 6/6 or better; (5) baseline axial length between 22 and 26 mm. The exclusion criteria were as follows: (1) history of myopia control interventions; (2) ocular abnormalities or systemic disease; (3) incomplete data; (4) incompliance to the treatment; (5) follow-up time of less than 365 ± 30 days.

### 2.2. Measurements and Follow-Up

All patients attending the clinic, including the ones in the present study group, undergo a full baseline ophthalmological assessment before commencing myopia control. After symptoms and history were taken, the ophthalmological examination included best corrected visual acuity (BCVA) with prestudy spectacles, cycloplegic refractive measurement, eye movement evaluation, cover test, prism cover-uncover tests for near and distance, slit-lamp evaluation, fundus examination and ocular biometry. 

Monocular decimal BCVA was tested using a Snellen chart. 

Cycloplegic refraction was measured at 30 min after the instillation of the last drop from 3 of the cyclopentolate 1% spaced at 5 min (Tonoref III Autorefractometer, Nidek, Aichi, Japan). For difficult dilatation and dark irides, tropicamide 1% and/or phenylephrine 2.5% were used. Cycloplegia was considered at a minimum of 6 mm pupil diameter or the absence of the pupillary light reflex [41].

Myopia was considered for SE of −0.50 D or less. Taking into account the refraction measurements, myopia was defined as low (−0.50 to −2.99 D), medium (−3.00 to −5.99 D) and high (−6.00 D or greater). 

SER was calculated as the average sphere + 1/2 cylinder value [1].

Ocular biometry was performed using ARGOS optical biometer (MOVU Inc., Komaki, Japan).

After myopia was diagnosed, the parents or caregivers of the suitable patients were provided with information on options for its control, including DIMS or SV lenses. The parents/caregivers were free to choose their preferred control method. The patients were instructed to wear the spectacles 10–12 h a day (with exceptions, such as swimming or bathing), and compliance with the treatment was checked at follow-up meetings.

The baseline ophthalmological examination was followed by two follow-ups at six-month and one-year intervals. On the occasion of each visit, BCVA, SER error after cycloplegia and AL were measured. For myopia progression greater than 0.5 D, lenses were replaced.

### 2.3. Statistical Analysis

The study was preceded by a sample size calculation. A minimum of 50 participants per group was necessary in order to achieve a power of 0.8 and a 5% significance level (α = 0.05), to detect a ±0.25 D difference in myopia progression between the two groups. 

Descriptive data are reported as mean ± SD, followed by the median and the range for the continuous variables. Absolute and relative frequencies were used for the categorical variables. Normality was assessed with the Shapiro–Wilk test. For normally distributed data, variables were compared using a paired-samples *t*-test; otherwise, the Wilcoxon test was employed. Chi-square test was used to evaluate categorical variables.

Boxplots were used in order to display the distribution of the SER and AL at the initial presentation, as well as at the two following meetings. The central line represents the median. The box represents the interquartile range, with the lower and upper boundaries of the box corresponding to the first (25th percentile) and third (75th percentile) quartiles. The whiskers extend to the extreme values within 1.5 times the interquartile range. The points beyond the whiskers represent potential outliers.

Multiple linear regression was carried out in order to model the linear relationship between predictors such as baseline SER and AL, treatment, age at intervention, sex and provenance. The RE was designated as the primary outcome. The corresponding results for the LE are provided in the Supplementary Materials section.

A significant statistical difference was set at *p* < 0.05. 

The statistical analysis was performed using the R software version 4.5.1 (R Core Team 2025, Vienna, Austria).

## 3. Results

**Figure 1:**
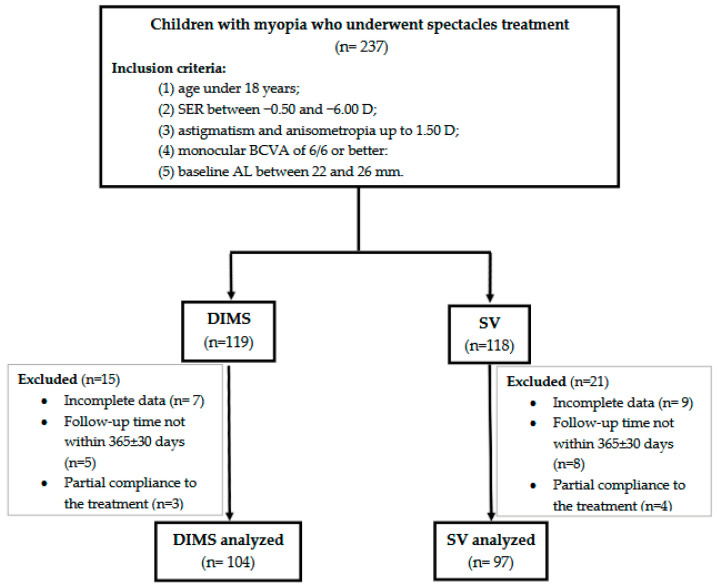
Flowchart of patient selection and study design. SER, spherical equivalent refraction; D, diopter; BCVA, best corrected visual acuity; AL, axial length; DIMS, defocus-incorporated multiple segment; SV, single vision.

The initial study included 237 patients satisfying the inclusion criteria. They were divided into two groups based on their treatment option: DIMS (n = 119) or SV (n = 118). A total of 36 patients were excluded due to incomplete data (n = 16), incomplete follow-up time (n = 13) or partial compliance with treatment (n = 7), as shown in Figure 1. 

A total of 201 children (mean age 10.92 ± 2.11 years, range, 7–17 years) were included in the final analysis, comprising 104 patients in the DIMS group and 97 in the SV group. The patients’ demographic data are presented in Table 1. A higher proportion of female patients was observed in the DIMS group (62.5%), whereas male children predominated in the SV group (53.61%). The other characteristics, including age, sex, provenance, SER and AL, were balanced between the two groups, with no statistically significant differences. The mean baseline SER values for the right eye were −1.89 ± 1.12 D in the DIMS group and −1.77 ± 1.01 D in the SV group (*p* = 0.9). The mean baseline AL values for the right eye were 24.11 ± 0.72 mm and 24.21 ± 0.58 mm in the DIMS and SV groups, respectively (*p* = 0.06).

**Figure 2:**
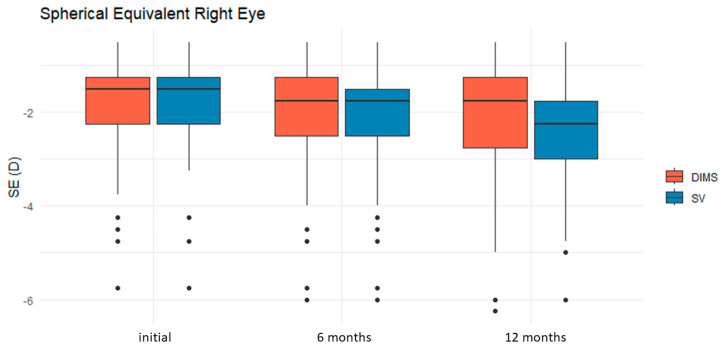
Changes in spherical equivalent refraction (SER) of the right eye over time and between the treatment groups. The distribution of the SER was broadly similar between the two groups at baseline. At 6 months, both groups showed a shift toward lower SER values, while maintaining comparable variability. At 12 months, the DIMS group showed a less myopic median SER than the SV group. The variability of the SER values was greater at 12 months in both groups, as reflected by the wider interquartile range and the whiskers.

**Figure 3:**
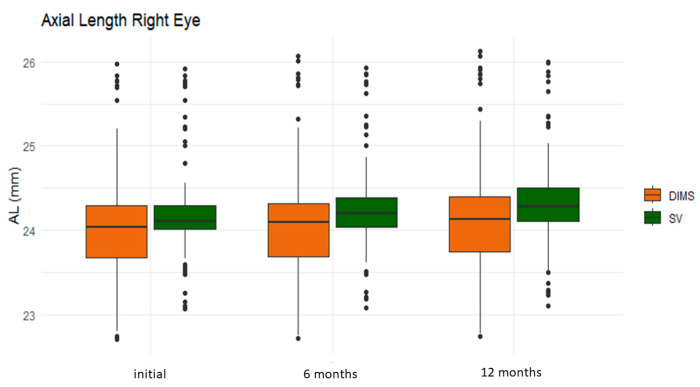
Changes in the axial length (AL) of the right eye over time and between the treatment groups. The distribution of the AL values was comparable between the DIMS and SV groups at baseline. Over the 12-month follow-up period, both groups demonstrated a gradual increase in AL, with slightly greater values observed in the SV group at 6 and 12 months. The interquartile ranges remained relatively stable throughout follow-up, indicating limited changes in within-group variability.

The mean SER progression in the right eye over the 12-month follow-up was −0.29 ± 0.3 D in the DIMS group and −0.58 ± 0.45 in the SV group, as shown in Table 2 and Table 3. Children wearing DIMS exhibited significantly less myopia progression (*p* < 0.001) (see Figure 2). The total increase in AL in the right eye over the study period was 0.09 ± 0.13 mm for the DIMS group and 0.14 ± 0.1 mm in the SV group (*p* < 0.01) (see Figure 3).

The multiple linear regression model evaluated the association between factors such as treatment option, age, gender, provenance and baseline SER and AL values and myopia progression for the RE. The model showed no significant effect of sex or baseline AL on SER change (*p* = 0.057 and 0.17, respectively), whereas treatment lens, age at diagnosis, provenance and baseline SER had a significant effect on SER change (*p* < 0.001 for all four factors) (see Table 4). The same factors were then evaluated for their association with AL change. Sex and baseline SER showed no effect on AL change (*p* = 0.23 and 0.65, respectively), whereas the treatment lens, age at diagnosis, provenance and baseline AL showed significant effects (*p* = 0.002, 0.04, <0.001 and <0.001, respectively). Similar results were obtained for the LE (see Table S1).

## 4. Discussion

The present study analyzed myopia progression and axial elongation in pediatric patients treated with DIMS versus SV lenses. Although myopia progressed in both groups over the 12-month evaluation period, as reflected by the SER values, the DIMS group demonstrated a more favorable outcome (−0.3 ± 0.29 D compared to −0.58 ± 0.45 D in the SV group, *p* < 0.001). Similar results were observed for AL, with a change of 0.11 ± 0.31 mm in the DIMS group compared to 0.15 ± 0.1 mm in the SV group. 

These results are consistent with previous findings in the literature. Lam et al. reported a 1-year SER change of −0.17 ± 0.05 D in the DIMS group compared to −0.55 ± 0.04 D in the SV group. Significant differences between the two lens types were also observed in the AL increase, with changes of 0.11 ± 0.02 mm versus 0.32 ± 0.02 mm, respectively. 

A retrospective observational study conducted in Turkey on 385 eyes analyzed the 12-month progression of myopia in three types of spectacles: SV (170 eyes, 29.4%), DIMS lenses (139 eyes, 38.2%) and Myopi-X progressive addition lenses (118 eyes, 32.4%). The results showed that mean SER progression was −0.35 ± 0.34 D for Myopi-X lenses, −0.46 ± 0.37 D for SV and −0.24 ± 0.33 D for DIMS (*p* < 0.001). Mean AL elongation was 0.21 ± 0.12 mm in the Myopi-X group, 0.24 ± 0.17 mm for the SV group and 0.17 ± 0.16 mm for the DIMS group (*p* = 0.004). The treatment effects on SER and AL changes were statistically significant (*p* < 0.001). Less myopic progression was observed with DIMS lenses, while the highest progression was observed in the SV group. The 12-month study concluded that DIMS lenses were the most effective in reducing refractive and axial progression and that baseline AL was the key parameter for axial growth [10]. 

Huang et al. also observed slower myopia progression in patients treated with DIMS lenses, although the 1-year changes were greater for both lens types compared to those reported in the present study. The SER change was 0.79 ± 0.47 D in the DIMS group and 1.07 ± 0.64 D in the SV group. It should be noted that the baseline SER values were higher in the Chinese study population, at 2.59 ± 1.11 D, compared to 1.81 ± 1.01 D in our study population. Similar findings were observed for AL, with changes of 0.41 ± 0.22 mm in the DIMS group and 0.52 ± 0.22 mm in the SV group. 

Differences in study outcomes may be attributable to variations in the populations evaluated. Recent cross-sectional studies reported substantial differences in the prevalence of myopia among children across ethnic groups, geographic regions and age groups, with these disparities becoming more pronounced among older school-aged children. For example, among Australian schoolchildren, the prevalence of myopia was 42.7% at 12 years of age and increased to 59.1% at 17 years among children of East Asian ancestry, compared with 8.3% and 17.7%, respectively, among European Caucasian children [42]. A cross-sectional study of American preschool children aged 6–72 months reported myopia prevalence rates of 1.2% in non-Hispanic White children, 3.7% in Hispanic children, 3.98% in Asian children, and 6.6% in African American children [43,44]. These findings highlight the potential influence of both ethnicity and age on myopia prevalence and development.

Previous studies have shown that children from eastern mainland China tend to experience an earlier onset of myopia and faster progression compared with children from Hong Kong. It has therefore been suggested that improved myopia control in these patients could be achieved by increasing the myopic defocus dose. Another possible explanation could be that higher AL values, likely resulting from rapid axial elongation, are more difficult to treat [9]. 

When examining the factors associated with SER and AL changes, the present study identified provenance, age at diagnosis, and baseline AL as statistically significant. Previous studies have analyzed the impact of age at diagnosis on treatment outcomes [9,25,28]. Neller et al. found that older children tended to respond more successfully to treatment, while Huang et al. reported a negative correlation. 

Lam et al., in a 6-year follow-up study, found that children who started treatment at an older age showed less axial elongation and slower myopia progression, whereas younger patients continued to progress more rapidly, despite DIMS lens wear [40]. A possible explanation may lie in differences in retinal profile or peripheral refraction [28]. It should also be noted that older children may be more compliant with spectacle wearing. Unlike previous research, the present study found a positive correlation with age. However, it should be noted that the minimum age in this study was 7 years, which is higher than that reported in other studies. 

A three-year, non-randomized, experimenter-masked retrospective controlled observational study of European individuals aged 6–16 years analyzed the SER and AL of myopic patients [45]. The patients were divided into four groups according to age (younger or older than 10 years) and lens type (DIMS or SV lenses). After three years of follow-up, the study reported reduced myopia progression in the DIMS groups compared with the SV groups. According to linear regression analysis, a significant correlation (*p* < 0.05) was observed between myopia progression (SER) and patient age in the DIMS groups, both in children younger and older than 10 years. Regarding AL, a significant linear regression was observed only in the DIMS group older than 10 years. The study concluded that DIMS lenses reduce the progression of myopia, especially in patients older than 10 years of age, and that AL measurement may be a more reliable parameter for evaluating myopic patients [45].

A study involving 396 myopic children evaluated the progression of myopia and astigmatism in patients wearing DIMS lenses (n = 192) and patients with SV lenses (n = 204). Changes in SE, diopter sphere, diopter cylinder, AL and corneal astigmatism were assessed over a one-year period. Myopia progression, as measured by SE, was significantly greater in the SV group (−0.95 ± 0.57 D, *p* < 0.001) than in the DIMS group (−0.30 ± 0.45 D). Although a small statistically significant difference in diopter cylinder (DIMS: −0.17 ± 0.28 D vs. SV: −0.20 ± 0.38 D, *p* = 0.01) was observed between the groups, changes in corneal astigmatism (DIMS: −0.19 ± 0.57 D vs. SV: −0.20 ± 0.66 D, *p* = 0.624) were comparable. The authors therefore concluded that DIMS lenses slow myopia progression without increasing astigmatism progression, suggesting that changes in astigmatism are more likely attributable to physiological and environmental factors influencing corneal shape.

When interpreting the results of the present study, several limitations must be considered. The first limitation is its retrospective nature. The absence of randomization, as parents selected the type of lens, may have introduced selection bias.

Another limitation of the study is the relatively short follow-up period. Nevertheless, Lam et al. reported sustained effects of DIMS lenses on SER and AL changes over a 3-year follow-up period.

A difference in sex distribution was observed between the two groups, with a predominance of females in the DIMS group. Sex has previously been evaluated as a potential factor associated with myopia progression. Recent studies have reported faster myopia progression among females, which may be attributable to differences in lifestyle behaviors, including greater time spent on near-work activities and less time engaged in outdoor activities [46]. Previous studies investigating DIMS spectacle lenses have not reported significant differences in treatment efficacy according to sex, suggesting that the myopia-control effect of DIMS lenses is likely consistent between boys and girls [38].

Although all the patients were followed for 12 months, variations in treatment compliance and environmental factors, such as outdoor activities, may have acted as confounding factors possibly affecting the results. The retrospective design of the present study and the absence of detailed data on outdoor and near-work activities, which prevented their quantification, represent additional limitations. Although the contribution of near-work activities as a risk factor for myopia remains poorly defined in the literature, increased time spent on indoor activities has been associated with a higher risk of myopia. Sustained accommodation resulting from continuous reading, longer reading durations and shorter reading distances have been suggested to promote ocular growth [47].

## 5. Conclusions

To the best of our knowledge, this is the first published article concerning the efficacy of DIMS spectacle lenses on the myopia progression in a Romanian pediatric population. The results of our study showed that DIMS lenses are an effective non-invasive treatment in myopia therapeutic management in the Caucasian population.

The progression of myopia is influenced by ethnicity, genetics, age of myopia onset and environmental factors such as near work, lack of outdoor activities, and education. All of these are key factors in the progression of myopia. In order to analyze the progression of myopia, all of the factors mentioned above should be taken into account.

## Figures and Tables

**Table 1:** Demographics for the study groups. Data are reported as mean ± SD, followed by the median and the range between brackets.

	Total (n = 201)	DIMS (n = 104)	SV (n = 97)	*p*-Value
Gender				
Female	110 (54.73%)	65 (62.5%)	45 (46.39%)	0.03
Male	91 (45.27%)	39 (37.5%)	52 (53.61%)	
Age	10.92 ± 2.11	10.9 ± 2.21	10.94 ± 2	0.91
	10 (7–17)	10 (7–17)	11 (7–17)	
Provenance				
Urban	117	60	57	0.99
Rural	84	44	40	
SER RE (D)	−1.83 ± 1.07	−1.89 ± 1.12	−1.77 ± 1.01	0.95
	−1.5 (−5.75 to −0.5)	−1.5 (−5.75 to −0.5)	−1.5 (−5.75 to −0.5)	
SER LE (D)	−1.81 ± 1.01	−1.86 ± 1.09	−1.77 ± 0.92	0.9
	−1.75 (−5.75 to −0.5)	−1.75 (−5.75 to −0.5)	−1.75 (−5.75 to −0.5)	
AL RE (mm)	24.16 ± 0.66	24.11 ± 0.72	24.22 ± 0.58	0.06
	24.1 (22.7 to 25.98)	24.04 (22.7 to 25.98)	24.11 (23.06 to 25.92)	
AL LE (mm)	24.15 ± 0.64	24.1 ± 0.73	24.21 ± 0.53	0.09
	24.14 (22.25 to 25.93)	24.06 (22.25 to 25.93)	24.15 (23 to 25.93)	

**Table 2:** Summary of the refractive (SER) and biometric (AL) parameters for each study group at baseline, 6 months and 12 months.

	Initial		At 6 Months		At 12 Months	
	DIMS (n = 104)	SV (n = 97)	DIMS (n = 104)	SV (n = 97)	DIMS (n = 104)	SV (n = 97)
SER RE (D)	−1.89 ± 1.12 −1.5 (−5.75 to −0.5)	−1.77 ± 1.01 −1.5 (−5.75 to −0.5)	−1.99 ± 1.14 −1.75 (−6 to −0.5)	−2.02 ± 1 −1.75 (−6 to −0.5)	−2.18 ± 1.16 −1.75 (−6.25 to −0.5)	−2.35 ± 1.04 −2.25 (−6 to −0.5)
SER LE (D)	−1.86 ± 1.09 −1.75 (−5.75 to −0.5)	−1.77 ± 0.92 −1.75 (−5.75 to −0.5)	−1.96 ± 1.12 −1.75 (−5.75 to −0.5)	−2.02 ± 0.95 −2 (−6 to −0.5)	−2.15 ± 1.16 −1.88 (−6 to −0.5)	−2.35 ± 1.01 −2.25 (−6 to −0.75)
AL RE (mm)	24.11 ± 0.72 24.04 (22.7 to 25.98)	24.22 ± 0.58 24.11 (23.06 to 25.92)	24.13 ± 0.72 24.09 (22.72 to 26.07)	24.28 ± 0.57 24.2 (23.08 to 25.93)	24.2 ± 0.74 24.13 (22.74 to 26.13)	24.36 ± 0.58 24.28 (23.1 to 26)
AL LE (mm)	24.1 ± 0.73 24.06 (22.25 to 25.93)	24.21 ± 0.53 24.15 (23 to 25.93)	24.12 ± 0.73 24.08 (22.26 to 25.95)	24.28 ± 0.53 24.23 (23.12 to 25.95)	24.21 ± 0.77 24.15 (22.26 to 26.65)	24.36 ± 0.54 24.32 (23.14 to 26)

**Table 3:** Differences in SE and AL for each study group at 6 months and 12 months (mean ± sd) by comparison to baseline.

	At 6 Months			At 12 Months		
	DIMS (n = 104)	SV (n = 97)	*p*-Value *	DIMS (n = 104)	SV (n = 97)	*p*-Value *
SER RE (D)	−0.1 ± 0.15	−0.25 ± 0.26	<0.001	−0.29 ± 0.3	−0.58 ± 0.45	<0.001
SER LE (D)	−0.1 ± 0.16	−0.25 ± 0.27	<0.001	−0.3 ± 0.29	−0.58 ± 0.45	<0.001
AL RE (mm)	0.02 ± 0.12	0.06 ± 0.06	<0.001	0.09 ± 0.13	0.14 ± 0.1	<0.001
AL LE (mm)	0.03 ± 0.05	0.07 ± 0.05	<0.001	0.11 ± 0.31	0.15 ± 0.1	<0.001

* comparison of the differences between the 2 study groups.

**Table 4:** Factors associated with changes in SER and AL for the right eye during the 1-year follow-up using multiple linear regression (statistical significance was set at 0.05).

	SER at 1 Year			AL at 1 Year		
	β	95% CI	*p*-Value	β	95% CI	*p*-Value
DIMS vs SV	−0.289	(−0.389, −0.189)	<0.001	0.051	(0.019. 0.084)	0.002
Age at diagnosis	0.043	(0.019, 0.067)	<0.001	−0.008	(−0.016, −0.0005)	0.04
Sex (male vs. female)	0.098	(−0.003, 0.201)	0.057	−0.019	(−0.053, −0.013)	0.23
Provenance (urban vs rural)	−0.213	(−0.313, −0.113)	<0.001	0.057	(0.025, 0.089)	<0.001
Baseline SER	0.957	(0.888, 1.025)	<0.001	0.005	(−0.017, 0.027)	0.65
Baseline AL	−0.078	(−0.190, 0.034)	0.17	1.014	(0.978, 1.05)	<0.001

Abbreviations: SER, spherical equivalent refraction; AL, axial length; CI, confidence interval; DIMS, defocus-incorporated multiple segment; SV, single vision.

## Data Availability

The data of this study are available upon request from the corresponding author. The data are not publicly available due to institutional data protection policies and to protect patient confidentiality.

## Supplementary Materials

The following supporting information can be downloaded at: https://www.mdpi.com/article/10.3390/bioengineering13070802/s1, Table S1: Factors associated with changes in SER and AL for the left eye during the 1 year follow-up using multiple linear regression (statistical significance was set at 0.05).

## Abbreviations

The following abbreviations are used in this manuscript:

DIMS	Defocus-incorporated multi-segment spectacle lens
SV	Single-vision
BCVA	Best corrected visual acuity
SER	Spherical equivalent
VA	Visual acuity
AL	Axial length
ATP	Atropine
COMET	Correction of Myopia Evaluation Trial
D	Diopters
PAL	Progressive addition lens
RE	Right eye
LE	Left eye
