# Supplementary material for: Defocus-Incorporated Multiple-Segment (DIMS) Spectacle Lenses Versus Single-Vision Lenses for Myopia Control in a South-Eastern European Population
Source: Bioengineering (Basel). 2026 Jul 13;13(7):802. doi: 10.3390/bioengineering13070802 (PMC13405324; doi:10.3390/bioengineering13070802)
Supplement: Supplementary file 1 [file bioengineering-13-00802-s001.zip › bioengineering-4380327-supplementary.pdf]

bioengineering

Supplementary Materials

Table S1 – Factors associated with changes in SER and AL for the left eye during the 1 year follow-up using multiple linear regression (statistical significance was set at 0.05)

	SER at 1 year			AL at 1 year		
	**β**	**95% CI**	***p*-value**	**β**	**95% CI**	***p*-value**
**DIMS vs SV**	-0.299	(-0.395, -0.203)	<0.001	0.049	(-0.018. 0.116)	0.15
**Age at diagnosis**	0.059	(0.035, 0.083)	<0.001	-0.015	(-0.032, 0.001)	0.07
**Sex (male vs. female)**	0.112	(0.014, 0.209)	0.03	-0.05	(-0.118, 0.018)	0.15
**Provenance (urban vs rural)**	-0.196	(-0.293, -0.1)	<0.001	0.012	(-0.056, 0.079)	0.7
**Baseline SER**	1.03	(0.96, 1.1)	<0.001	-0.021	(-0.07, 0.027)	0.4
**Baseline AL**	-0.029	(-0.136, 0.079)	0.6	0.971	(0.895, 1.046)	<0.001

Abbreviations: SER, spherical equivalent refraction; AL, axial length, CI, confidence interval; DIMS, defocus-incorporated multiple segments; SV, single vision.
